# Editorial: Bunyaviruses - threats to health and economy

**DOI:** 10.3389/fcimb.2024.1369530

**Published:** 2024-02-02

**Authors:** Wei Ye, Feihu Yan

**Affiliations:** ^1^ Department of Microbiology, School of Preclinical Medicine, Airforce Medical University (Fourth Military Medical University), Xi’an, Shaanxi, China; ^2^ Key Laboratory of Jilin Province for Zoonosis Prevention and Control, Changchun Veterinary Research Institute, Chinese Academy of Agricultural Sciences, Changchun, Jilin, China

**Keywords:** Bunyavirales, Hantaviridae, Phenuiviridae, Nairoviridae, Dabie bandavirus (DBV), severe fever with thrombocytopenia syndrome virus (SFTSV), Rift Valley fever virus (RVFV), Crimean–Congo hemorrhagic fever virus (CCHFV)

Previously, Bunyaviridae, which was upgraded to Bunyavirales after the 10th International Committee on Taxonomy of Viruses (ICTV) (Maes et al., 2018). Under the new virus taxonomy system, the arenaviridae has also been included in the bunyavirales, which means that bunyaviruses contain the most diverse pathogenic viruses that are detrimental to both animals and plants (Maes et al., 2018). The increase in global human activity and rapid urbanization has increased the possibility of human contact with wildlife. Most bunyaviruses originate from zoonotic diseases, and outbreaks may result in significant loss of life, economic contraction, and social instability.

In this Research Topic, Chen et al. generalized the current status of the development of three highly pathogenic bunyavirus vaccines, with a focus on Crimean–Congo hemorrhagic fever virus, Rift alley fever Virus (RVFV), and Hantaan Virus (HTNV), characterized by Nairoviridae, Phenuiviridae, and Hantaviridae, respectively. At present, in addition to the HFRS inactivated vaccine used in East Asia, CCHFV inactivated vaccines are derived from mouse brains and used in Bulgaria, but there are no other vaccines available for human use against Bunyaviruses. This review summarizes the current status of vectored-, subunit-, and DNA-vaccine development and proposes new directions for these viruses, including improved subunit vaccines and mRNA-LNP-based vaccines.

RVF is an arboviral disease that is transmitted mainly through mosquito bites. Since its discovery in the Great Rift Valley of East Africa, the prevalence of RVFV has been continuously expanding, with the most noteworthy being the outbreak in Egypt in 1977 and its entry into the Arabian Peninsula in 2001 (Linthicum et al., 2016). The research conducted by Bron et al. focused on the transmission dynamics of RVFV infection in natural hosts through artificial reconstruction. They found that lambs infected with RVFV required more bites to ensure viremia, and a peak in viral load occurred 2-4 days after biting. Their results indicate that the likelihood of a successful infection in ruminant hosts is influenced by the number of infectious mosquito bites, with an estimated transmission efficiency of 28% (95% confidence interval: 15-47%) for each bite. The results of using mathematical models to simulate RVFV outbreaks in lambs (i.e., R0>1) indicate the required host-to-mosquito ratio and provide a reference for proposing targeted prevention strategies in the future.

In addition to epidemiology, from a molecular biology perspective, the replication and assembly of a virus itself are extremely important for maintaining its life cycle. In this Research Topic, Tercero et al. found multiple viral RNA binding sites within Gn via UV-crosslinking and immunoprecipitation followed by high-throughput sequencing analysis (CLIP-seq analysis). These modifications are important for the efficient packaging of antigenomic S RNA into virions. Among these sites, one located in the 3’ noncoding region was important for the ability of the virus to suppress interferon-β signaling. These findings suggest the important role of the interaction between viral RNA and structural glycoproteins in the RVFV life cycle.

Severe fever with thrombocytopenia syndrome virus (SFTSV), a newly discovered tick-borne bunyavirus, has attracted increased amounts of attention because of its high mortality rate of more than 40% (Li et al., 2021). In the recent ICTV category, SFTSV was named Dabie bandavirus (DBV) of the Bandavirus genus within the Phenuiviridae family (Sasaya et al., 2023). In this study, Song et al. retrospectively analyzed the clinical outcomes of SFTSV-infected patients under co-infected conditions. They found that, out of 157 infected patients, 43 patients with co - infections had a higher mortality rate compared to 114 non - co - infected patients did (P=0.011). Therefore, appropriate treatment strategies are needed to prevent co infections, and strengthened monitoring and timely and appropriate treatment are needed to minimize mortality rates.

Although bunyaviruses cause severe diseases in humans, the specific pathogenic mechanism involved is largely unknown. In addition to direct virus-induced injury, immunological injury is considered to play an important role in bunyavirus infection-directed pathogenesis (Brocato and Hooper, 2019). Among these bunyaviruses, hantavirus infection does not cause an obvious cytopathic effect, and cytotoxic T lymphocyte responses have been shown to play a role in hantavirus-induced diseases (Noack et al., 2020). In this Research Topic, Zhang et al. revealed that during Hantaan virus (HTNV) infection, CD8+ T cells were bystander activated after HTNV infection in HFRS patients and may be involved in host injury. Mechanistically, HTNV-infected endothelial cells produced high levels of IL-15, which was positively correlated with disease severity and the expression of NKG2D on bystander-activated CD8+ T cells. Moreover, elevated IL-15 could induce the activation of IL-15Rβ+NKG2D+ bystander CD8+ T cells. The expression of IL-15Rα and its ligand NKG2D was upregulated in HTNV-infected endothelial cells. Bystander-activated CD8+ T cells can exert cytotoxic effects on endothelial cells, which can be enhanced by IL-15 stimulation and blocked by an NKG2D antibody. Therefore, the IL-15/NKG2D axis could serve as an important pathway for bystander activation of memory CD8+ T cells, which may be a possible mechanism for the pathogenesis of HFRS. However, whether the IL15/NKG2D axis contributes to bystander activation of CD8+ T cells in other human viral diseases and whether IL-15 or NKG2D could be considered therapeutic targets in the treatment of the immunopathology of viral diseases are questions worth exploring in the future.

Although the above articles in the Research Topic provide new interesting information on bunyaviruses, specific treatment choices, preventive vaccines and pathogenic mechanisms for these highly pathogenic viruses still need further investigation. In addition, epidemiological research and replication mechanisms may aid in antiviral development and herd management strategy optimization.

## Author contributions

WY: Writing – original draft, Writing – review & editing, Visualization. FY: Writing – review & editing, Supervision, Visualization.
